# Dysregulation of lncRNAs in autoimmune neuropathies

**DOI:** 10.1038/s41598-021-95466-w

**Published:** 2021-08-09

**Authors:** Mahdi Gholipour, Mohammad Taheri, Jafar Mehvari Habibabadi, Naghme Nazer, Arezou Sayad, Soudeh Ghafouri-Fard

**Affiliations:** 1 Department of Medical Genetics, School of Medicine, Shahid Beheshti University of Medical Sciences, Tehran, Iran; 2 Skull Base Research Center, Loghman Hakim Hospital, Shahid Beheshti University of Medical Sciences, Tehran, Iran; 3 Isfahan Neuroscience Research Center, Isfahan University of Medical Sciences, Isfahan, Iran; 4 Department of Electrical Engineering, Sharif University of Technology, Tehran, Iran

**Keywords:** Genetics, Immunology, Molecular biology, Neuroscience

## Abstract

Chronic inflammatory demyelinating polyradiculoneuropathy (CIDP) and Guillain-Barré syndrome (GBS) are inflammatory neuropathies with different clinical courses but similar underlying mechanisms. Long non-coding RNAs (lncRNAs) might affect pathogenesis of these conditions. In the current project, we have selected *HULC*, *PVT1*, *MEG3*, *SPRY4-IT1*, *LINC-ROR* and *DSCAM-AS1* lncRNAs to appraise their transcript levels in the circulation of CIDP and GBS cases versus controls. Expression of *HULC* was higher in CIDP patients compared with healthy persons (Ratio of mean expression (RME) = 7.62, SE = 0.72, P < 0.001). While expression of this lncRNA was not different between female CIDP cases and female controls, its expression was higher in male CIDP cases compared with male controls (RME = 13.50, SE = 0.98, P < 0.001). Similarly, expression of *HULC* was higher in total GBS cases compared with healthy persons (RME = 4.57, SE = 0.65, P < 0.001) and in male cases compared with male controls (RME = 5.48, SE = 0.82, P < 0.001). Similar pattern of expression was detected between total cases and total controls. *PVT1* was up-regulated in CIDP cases compared with controls (RME = 3.04, SE = 0.51, P < 0.001) and in both male and female CIDP cases compared with sex-matched controls. Similarly, *PVT1* was up-regulated in GBS cases compared with controls (RME = 2.99, SE = 0.55, P vale < 0.001) and in total patients compared with total controls (RME = 3.02, SE = 0.43, P < 0.001). Expression levels of *DSCAM-AS1* and *SPRY4-IT1* were higher in CIDP and GBS cases compared with healthy subjects and in both sexes compared with gender-matched healthy persons. Although *LINC-ROR* was up-regulated in total CIDP and total GBS cases compared with controls, in sex-based comparisons, it was only up-regulated in male CIDP cases compared with male controls (RME = 3.06, P = 0.03). Finally, expression of *MEG3* was up-regulated in all subgroups of patients versus controls except for male GBS controls. *SPRY4-IT* could differentiate CIDP cases from controls with AUC = 0.84, sensitivity = 0.63 and specificity = 0.97. AUC values of *DSCAM-AS1*, *MEG3*, *HULC*, *PVT1* and *LINC-ROR* were 0.80, 0.75, 0.74, 0.73 and 0.72, respectively. In differentiation between GBS cases and controls, *SPRY4-IT* and *DSCAM-AS1* has the AUC value of 0.8. None of lncRNAs could appropriately differentiate between CIDP and GBS cases. Combination of all lncRNAs could not significantly enhance the diagnostic power. Taken together, these lncRNAs might be involved in the development of CIDP or GBS.

## Introduction

Chronic inflammatory demyelinating polyradiculoneuropathy (CIDP) and Guillain-Barré syndrome (GBS) are inflammatory neuropathies with different clinical courses. While CIDP has a slowly progressive onset^1^, GBS has an acute-onset with ascending pattern of neuropathy^2^. Both conditions are associated with dysregulation of immune response^3,4^. In GBS, such responses are believed to be triggered by infectious conditions in the respiratory or gastrointestinal tract leading to a functional failure in the blood–nerve barrier and damage of myelin sheaths and/or nerve fibers^5^. Almost all aspects of immune function including humoral responses, complement, T cells and macrophages participate in the pathogenesis of these immune-mediated neuropathies^6^. However, the underlying cause of such extensive immune dysregulation is not thoroughly identified^3^. Long non-coding RNAs (lncRNAs) have central influences on the activity of immune system^7,8^. Contribution of a number of these transcripts in the pathogenesis of CIDP and GBS has been recently verified by our group^9^. However, the role of several members of lncRNAs in autoimmune neuropathies needs to be elucidated. In the current project, we have selected *HULC*, *PVT1*, *MEG3*, *SPRY4-IT1*, *LINC-ROR* and *DSCAM-AS1* lncRNAs to appraise their transcript levels in the circulation of CIDP and GBS cases versus controls. The reason for selection of these lncRNAs was their roles in modulation of immune responses. *HULC* has been identified as one of important factors in induction of pro-inflammatory responses in the course of liposaccharide-associated sepsis in endothelial cells^10^. *Pvt1* has been shown to modulate the immunosuppression function of granulocytic myeloid-derived suppressor cells in animal models^11^. *MEG3* has been reported to induce imbalance between regulatory T cells and Th17 cells^12^. *SPRY4-IT1* interacts with ERRα^13^, a nuclear receptor which regulates innate immunity^14^. *LINC-ROR* has functional interaction with TGF-β to regulated hypoxia-induced cellular cascades^15^. Finally, *DSCAM-AS1* has been shown to regulate several genes which are implicated in inflammatory responses^16^. These lncRNAs regulate immune reactions via different routes.

## Materials and methods

### Recruitment of GBS/CIDP cases and normal controls

A total of 32 CIDP patients with typical type (11 females, 21 males), 25 GBS patients (7 females, 18 males), and 58 healthy individuals (20 females and 38 males) participated in the current investigation. CIDP cases had symmetric muscle weakness which affected both proximal and distal muscles. The course of disorder was compliant with a motor-predominant neuropathy. Patients were assessed using the guidelines stated by American Academy of Neurology^17^ and National Institute of Neurological Disorders and Stroke^18^. In addition, electrophysiological criteria were used for diagnosis of GBS^19^. Blood samples were obtained when patients entered the remission phase and were not on any treatment. All were responsive to corticosteroids or IVIg treatment. No concomitant treatment was used for these patients. None of them had any comorbid condition. Persons recruited as controls had no recent or chronic infection, malignant condition, or any systemic diseases. The study protocol was approved by the ethical committee of Shahid Beheshti University of Medical Sciences (IR.SBMU.MSP.REC.1399.575) and the study protocol is performed in accordance with the relevant guidelines. Informed consent forms were signed by all recruited persons.

### Expression assay

Three milliliters of the peripheral blood of all recruited people were obtained for RNA extraction. This phase was performed using the GeneAll kit (Seoul, Korea). The retrieved RNA was then transformed to cDNA using the kit prepared by the Thermo Fisher Scientific Company (Brussels, Belgium). Expression levels of mentioned lncRNAs were measured in GBS and CIDP cases versus healthy persons using the Ampliqon master mix (Odense, Denmark). Reactions were executed in the Step One Plus Real-Time PCR system (Applied Biosystems, Foster city, CA, USA). Table 1 shows the characteristics of primers designed for amplification of *HULC*, *PVT1*, *MEG3*, *SPRY4-IT1*, *LINC-ROR* and *DSCAM-AS1*.

**Table 1:** Characteristics of primers designed for amplification of *HULC*, *PVT1*, *MEG3*, *SPRY4-IT1*, *LINC-ROR* and *DSCAM-AS1*.

Gene	Primer sequence		Primer length	Product size
HULC	Forward primer	ACGTGAGGATACAGCAAGGC	20	75
	Reverse primer	AGAGTTCCTGCATGGTCTGG	20	
PVT1	Forward primer	CCCATTACGATTTCATCTC	19	131
	Reverse primer	GTTCGTACTCATCTTATTCAA	21	
MEG3	Forward primer	TGGCATAGAGGAGGTGAT	18	111
	Reverse primer	GGAGTGCTGTTGGAGAATA	19	
SPRY4-IT1	Forward primer	AGCCACATAAATTCAGCAGA	20	115
	Reverse primer	GATGTAGGATTCCTTTCA	18	
LINC-ROR	Forward primer	TATAATGAGATACCACCTTA	20	170
	Reverse primer	AGGAACTGTCATACCGTTTC	20	
DSCAM-AS1	Forward primer	TCAGTGTCGCTACAGGGGAT	20	118
	Reverse primer	GGAGGAGGGACAGAGAAGGA	20	
B2M	Forward primer	AGATGAGTATGCCTGCCGTG	20	105
	Reverse primer	GCGGCATCTTCAAACCTCCA	20	

### Statistical methods

Expression of selected lncRNAs were analyzed in the R V.34 software^20^. Transcript magnitudes of these lncRNAs in comparison with the levels of *B2M* gene were measured from Ct and efficiency values. The obtained figures were log2 transformed. The significance of difference in mean values of transcript intensities of lncRNAs was judged using the t-test. Correlations between expression quantities were appraised using Spearman correlation test. Receiver operating characteristic (ROC) curves were plotted to quantify the diagnostic values of expression levels of lncRNAs. Youden's J statistic was used to determine the optimum threshold. Area under curve (AUC) values were quantified.

### Ethics approval and consent to participant

All procedures performed in studies involving human participants were in accordance with the ethical standards of the institutional and/or national research committee and with the 1964 Helsinki declaration and its later amendments or comparable ethical standards. Informed consent forms were obtained from all study participants. The study protocol was approved by the ethical committee of Shahid Beheshti University of Medical Sciences (IR.SBMU.MSP.REC.1399.575). All methods were performed in accordance with the relevant guidelines and regulations.

## Results

Table 2 demonstrates demographic and clinical data of patients.

**Table 2:** Demographic and clinical data of patients.

	GBS	CIDP
Age (mean ± SD, Y)	49.72 ± 14.6	50.5 ± 15.8
Prolonged F waves (%)	70	90
Prolonged distal motor latency (%)	58	85
Slowed conduction velocity (%)	67	87
Conduction block (%)	30	65

Expression of *HULC* was higher in CIDP patients compared with controls (Ratio of mean expression (RME) = 7.62, SE = 0.72, P < 0.001). While expression of this lncRNA was similar between female CIDP cases and female controls, its expression was up-regulated in male CIDP cases compared with male controls (RME = 13.50, SE = 0.98, P < 0.001). Similarly, expression of *HULC* was higher in total GBS cases compared with controls (RME = 4.57, SE = 0.65, P < 0.001) and in male cases compared with male controls (RME = 5.48, SE = 0.82, P < 0.001). Similar pattern of expression was detected between total cases and total controls. *PVT1* was up-regulated in CIDP cases compared with controls (RME = 3.04, SE = 0.51, P < 0.001) and in both male and female CIDP cases compared with sex-matched healthy persons. Similarly, *PVT1* was up-regulated in GBS cases compared with controls (RME = 2.99, SE = 0.55, P vale < 0.001) and in total patients compared with total controls (RME = 3.02, SE = 0.43, P < 0.001). Expression levels of *DSCAM-AS1* and *SPRY4-IT1* were higher in CIDP and GBS cases compared with controls and in both sexes compared with gender-matched healthy subjects. Although *LINC-ROR* was up-regulated in total CIDP and total GBS cases compared with controls, in sex-based comparisons, it was only up-regulated in male CIDP cases compared with male controls (RME = 3.06, P = 0.03). Finally, expression of *MEG3* was up-regulated in all subgroups of patients versus controls except for male GBS controls (Table 3).

**Table 3:** Detailed parameters of expression analysis of lncRNAs in patients and controls.

		SE	RME	P Value	95% CI		SE	RME	P Value	95% CI		SE	RME	P Value	95% CI	
		*HULC*					*PVT1*					*DSCAM-AS1*				
**CIDP/Control**																
**Total**	**32/58**	0.72	7.62	**0.00**	1.49	4.37	0.51	3.04	**0.00**	0.58	2.63	0.77	13.52	**0.00**	2.23	5.28
**F**	**11/20**	0.93	2.57	0.16	–0.56	3.28	0.58	2.99	**0.02**	0.35	2.81	1.08	6.94	**0.02**	0.58	5.01
**M**	**21/38**	0.98	13.50	**0.00**	1.77	5.74	0.73	3.07	**0.03**	0.16	3.08	1.02	19.19	**0.00**	2.21	6.31
**GBS/Control**																
**Total**	**25/58**	0.65	4.57	**0.00**	0.90	3.49	0.55	2.99	**0.01**	0.47	2.70	0.73	11.70	**0.00**	2.09	5.01
**F**	**7/20**	0.94	3.89	0.05	–0.03	3.95	0.76	3.31	0.06	–0.05	3.51	0.93	26.07	**0.00**	2.79	6.62
**M**	**18/38**	0.82	5.48	**0.00**	0.81	4.10	0.74	2.79	0.05	–0.02	2.98	0.95	8.66	**0.00**	1.20	5.03
**CIDP/GBS**																
**Total**	**32/25**	0.71	1.67	0.31	–0.70	2.17	0.62	1.02	0.97	–1.21	1.26	0.75	1.16	0.78	–1.30	1.72
**F**	**11/7**	0.97	0.66	0.55	–2.66	1.47	0.89	0.90	0.87	–2.09	1.79	0.92	0.27	0.06	–3.87	0.05
**M**	**21/18**	0.95	2.46	0.18	–0.63	3.23	0.81	1.10	0.87	–1.51	1.78	0.98	2.22	0.25	–0.84	3.14
**All Patients/Control**																
**Total**	**57/58**	0.60	6.09	**0.00**	1.43	3.79	0.43	3.02	**0.00**	0.74	2.45	0.65	12.69	**0.00**	2.38	4.95
**F**	**18/20**	0.80	3.02	0.06	–0.04	3.23	0.48	3.11	**0.00**	0.66	2.62	0.94	11.61	**0.00**	1.63	5.45
**M**	**39/38**	0.78	8.90	**0.00**	1.60	4.71	0.61	2.94	**0.01**	0.35	2.76	0.86	13.29	**0.00**	2.01	5.45

		SE	RME	P Value	95% CI		SE	RME	P Value	95% CI		SE	RME	P Value	95% CI	
		*SPRY4–IT1*					*LINC-ROR*					*MEG3*				
**CIDP/Control**																
**Total**	**32/58**	0.76	25.02	**0.00**	3.13	6.16	0.76	6.55	**0.00**	1.19	4.23	0.85	10.96	**0.00**	1.74	5.17
**F**	**11/20**	0.93	31.54	**0.00**	3.03	6.93	1.06	3.00	0.15	–0.58	3.76	1.22	13.12	**0.01**	1.13	6.30
**M**	**21/38**	1.06	22.14	**0.00**	2.34	6.59	1.02	9.86	**0.00**	1.26	5.35	1.12	9.96	**0.01**	1.04	5.59
**GBS/Control**																
**Total**	**25/58**	0.64	9.96	**0.00**	2.04	4.60	0.75	3.06	**0.03**	0.12	3.11	0.77	5.13	**0.00**	0.81	3.90
**F**	**7/20**	0.89	27.37	**0.00**	2.85	6.70	1.22	5.04	0.07	–0.26	4.93	0.96	23.12	**0.00**	2.45	6.61
**M**	**18/38**	0.86	6.36	**0.00**	0.94	4.39	0.95	2.58	0.16	–0.55	3.28	1.01	2.54	0.19	–0.70	3.39
**CIDP/GBS**																
**Total**	**32/25**	0.69	2.51	0.06	–0.05	2.70	0.72	2.14	0.14	–0.35	2.55	0.95	2.14	0.25	–0.81	3.00
**F**	**11/7**	1.07	1.15	0.85	–2.06	2.47	1.17	0.60	0.54	–3.29	1.80	1.34	0.57	0.55	–3.67	2.03
**M**	**21/18**	0.89	3.48	0.05	–0.01	3.61	0.91	3.82	**0.04**	0.09	3.78	1.23	3.92	0.12	–0.53	4.47
**All Patients/Control**																
**Total**	**57/58**	0.63	16.70	**0.00**	2.82	5.31	0.67	4.69	**0.00**	0.90	3.56	0.67	7.86	**0.00**	1.65	4.30
**F**	**18/20**	0.74	29.85	**0.00**	3.40	6.40	0.96	3.67	0.06	–0.08	3.83	0.90	16.36	**0.00**	2.20	5.87
**M**	**39/38**	0.87	12.45	**0.00**	1.89	5.38	0.89	5.31	**0.01**	0.63	4.19	0.89	5.30	**0.01**	0.64	4.17

**Figure 1:**
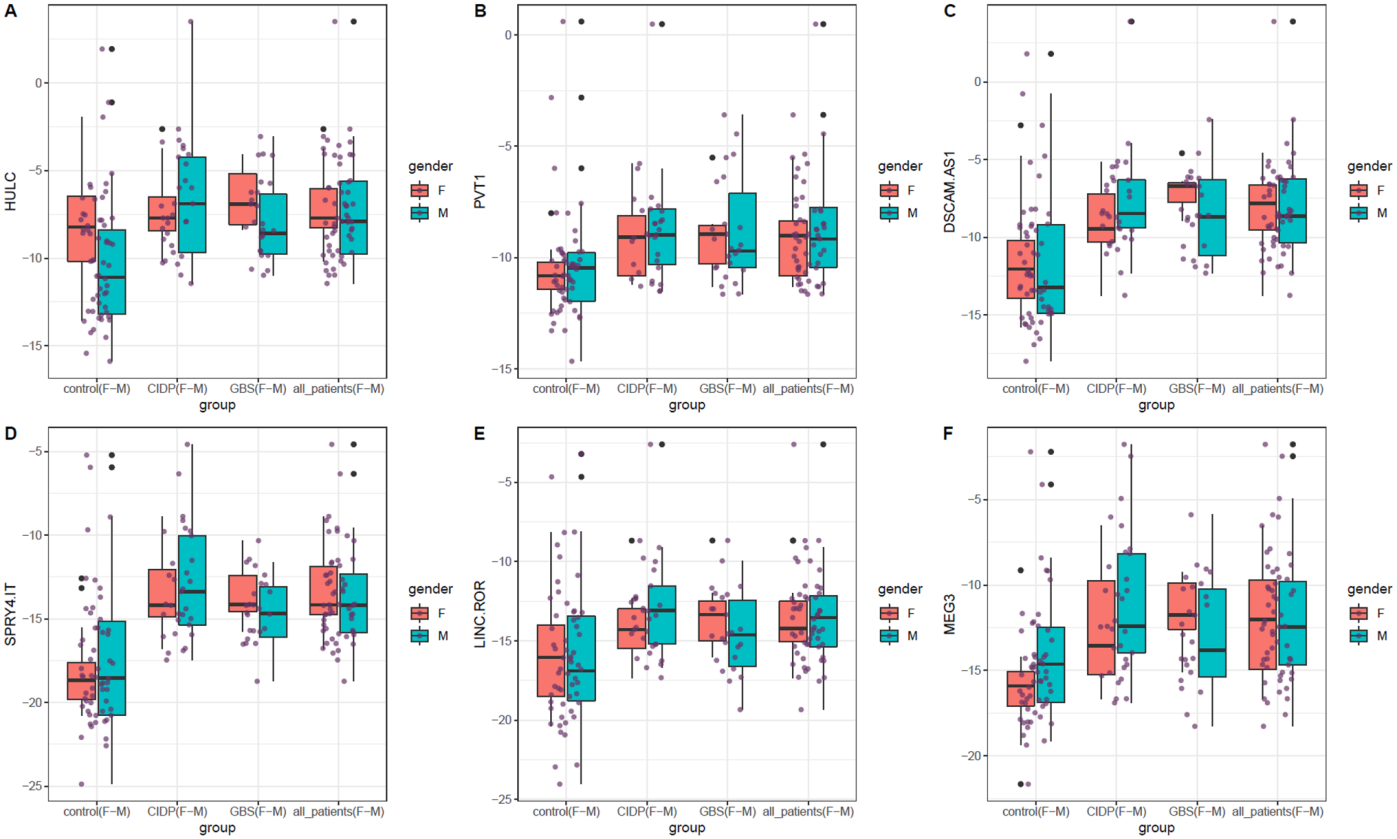
Expression levels of lncRNAs in study subgroups. Mean values and interquartile range are displayed. Purple dots show each expression level. Black dot represents outliers. (This figure has been depicted by R software)^20^.

Figure 1 displays expression amounts of selected lncRNAs in study subgroups.

**Figure 2:**
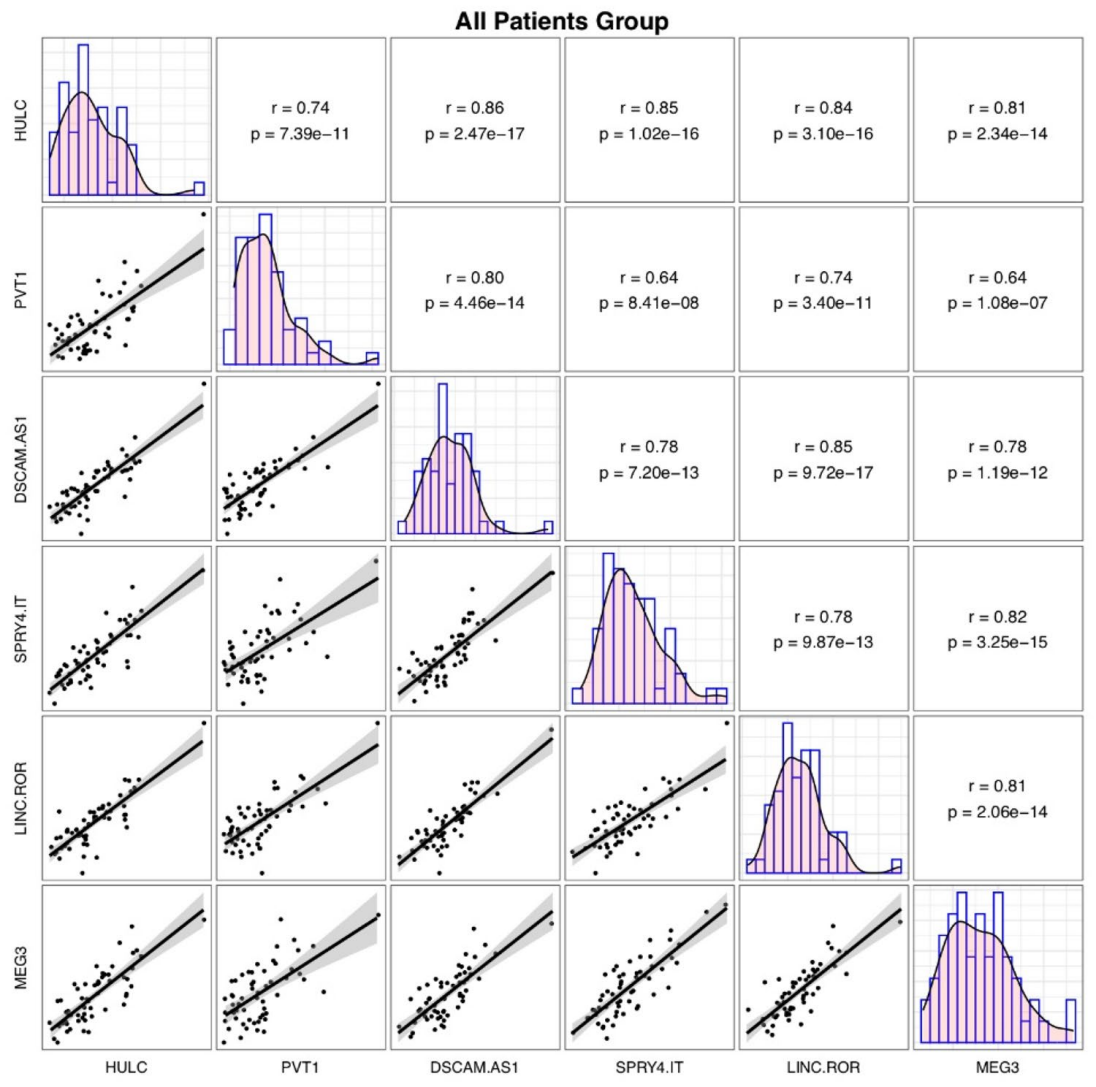
Correlations between expression quantities of lncRNAs among patients. (This figure has been depicted by R software)^20^.

Significant pairwise correlations have been identified between lncRNAs expressions with the most robust one being between *HULC*/*DSCAM-AS1* and *HULC*/*SPRY4-IT* pairs (r = 0.86 and 0.85 respectively) (Fig. 2).

**Figure 3:**
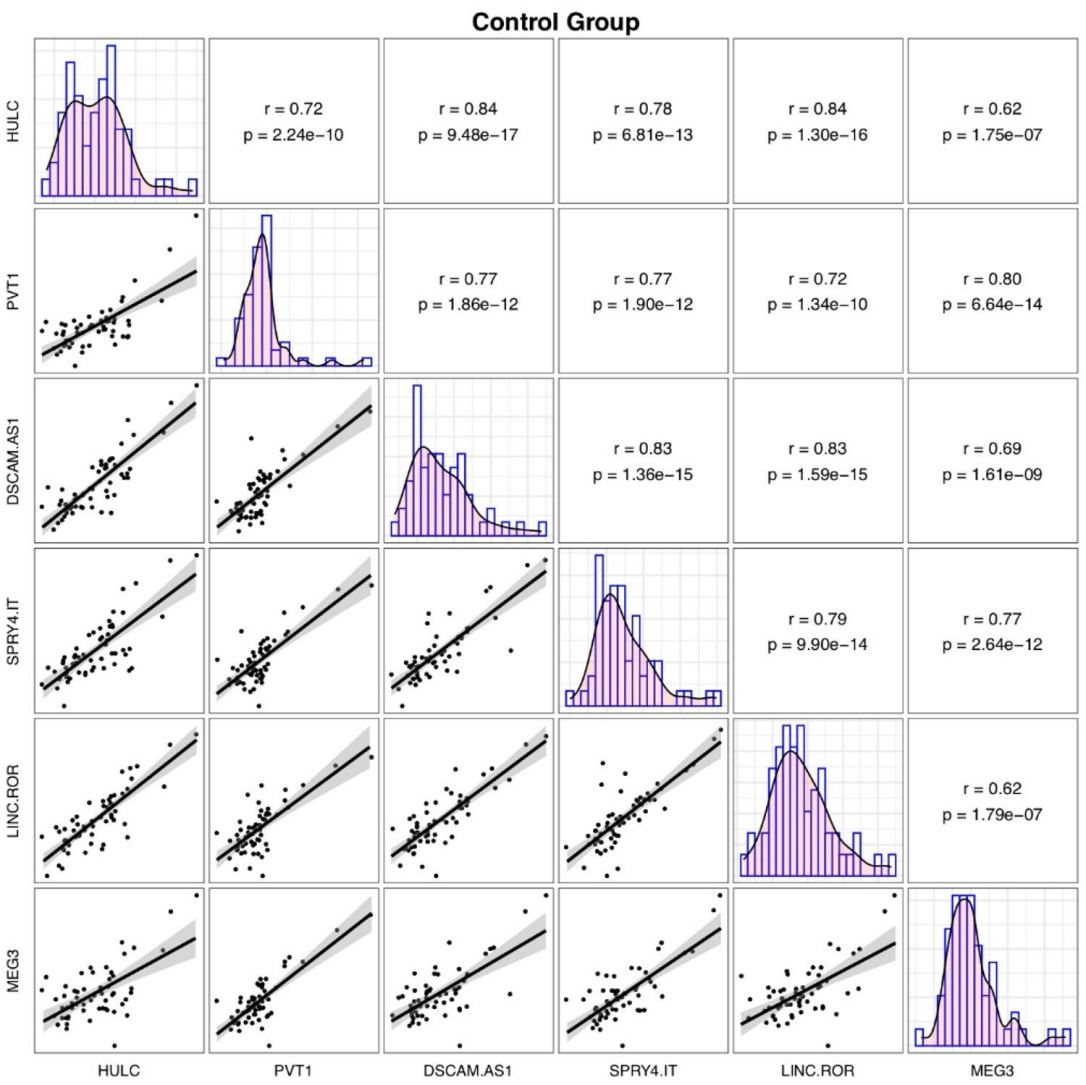
Correlations between expression quantities of lncRNAs among healthy controls. (This figure has been depicted by R software)^20^.

Among healthy controls, the most robust correlations have been reported between *HULC*/*DSCAM-AS1* and *HULC*/*LINC-ROR* pairs (r = 0.84 for both pairs) (Fig. 3).

**Figure 4:**
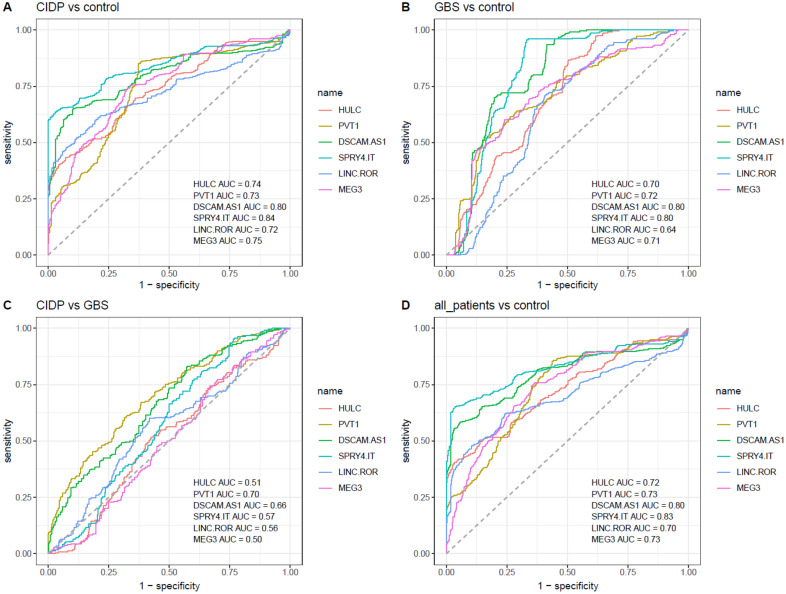
ROC curves showing the diagnostic power of lncRNAs in differentiation of CIDP cases from controls (**A**), GBS cases from controls (**B**), CIDP cases from GBS cases (**C**), and total patients from controls (**D**).

Finally, diagnostic power of lncRNAs for distinguishing patients from healthy subjects was assessed (Fig. 4).

*SPRY4-IT* could differentiate CIDP cases from controls with AUC = 0.84, sensitivity = 0.63 and specificity = 0.97. AUC values of *DSCAM-AS1*, *MEG3*, *HULC*, *PVT1* and *LINC-ROR* were 0.80, 0.75, 0.74, 0.73 and 0.72, respectively. In differentiation between GBS cases and controls, *SPRY4-IT* and *DSCAM-AS1* has the AUC value of 0.8. None of lncRNAs could appropriately differentiate between CIDP and GBS cases. Combination of all lncRNAs could not significantly enhance the diagnostic power (Table 4).

**Table 4:** Detailed parameters of depicted ROC curves.

Number of Samples		*HULC*			*PVT1*			*DSCAM-AS1*			*SPRY4-IT*			*LINC-ROR*			*MEG3*			All Markers		
		AUC	Sensitivity	Specificity	AUC	Sensitivity	Specificity	AUC	Sensitivity	Specificity	AUC	Sensitivity	Specificity	AUC	Sensitivity	Specificity	AUC	Sensitivity	Specificity	AUC	Sensitivity	Specificity
**CIDP/Control**																						
**Total**	**33/50**	0.74	0.43	0.93	0.73	0.85	0.63	0.80	0.65	0.89	0.84	0.63	0.97	0.72	0.60	0.80	0.75	0.74	0.67	0.79	0.60	0.88
**GBS/Control**																						
**Total**	**24/50**	0.70	0.87	0.49	0.72	0.52	0.84	0.80	0.94	0.58	0.80	0.96	0.67	0.64	0.71	0.59	0.71	0.60	0.76	0.78	0.91	0.58
**CIDP/GBS**																						
**Total**	**33/24**	0.51	0.52	0.58	0.70	0.60	0.68	0.66	0.83	0.43	0.57	0.96	0.23	0.56	0.60	0.58	0.50	0.74	0.33	0.59	0.63	0.59
**All Patients/Control**																						
**Total**	**57/50**	0.72	0.41	0.97	0.73	0.86	0.56	0.80	0.58	0.94	0.83	0.65	0.96	0.70	0.62	0.76	0.73	0.76	0.64	0.80	0.63	0.88

## Discussion

LncRNAs have been shown to take part in the pathogenesis of immune-related conditions. Up-regulation of lncRNAs has been reported in a number of these conditions. For instance, expression levels of *HOTAIR*, *LUST*, *anti-NOS2A*, *MEG9*, *SNHG4*, *TUG1*, and *NEAT1* have been shown to be increased in blood exosomes of patients with rheumatoid arthritis (RA) compared with exosomes retrieved from normal blood samples^21^. The same study has reported up-regulation of mentioned lncRNAs in addition to *H19 antisense*, *HAR1B* and *GAS5* in peripheral blood mononuclear cells of these patients^21^. *ENST00000483588* is another lncRNA which has been shown to be up-regulated in fibroblast-like synoviocytes of patients with RA^22^. A number of selected lncRNAs in the current project have been previously shown to be up-regulated in immune-mediated conditions. For instance, *PVT1* has been reported to be up-regulated in fibroblast-like synoviocytes of RA models parallel with down-regulation of sirt6, a putative target for this lncRNA. *PVT1* silencing or sirt6 over-expression could suppress cell proliferation and inflammation, while inducing cell apoptosis^23^. *MEG3* has been demonstrated to regulate RA pathogenesis through targeting NLRC5^24^. *LINC-ROR*, *MEG3*, *SPRY4-IT1* and *UCA1* have been among lncRNA with higher expression in patients with schizophrenia compared with normal subjects^25^.

CIDP and GBS disorders are two immune-mediated conditions in which lncRNAs might contribute. We measured expression of amounts of six immune-related lncRNAs in the circulation of these patients versus healthy controls. Expression of *HULC* was higher in CIDP patients compared with controls. While expression of this lncRNA was not different between female CIDP cases and female controls, its expression was higher in male CIDP cases compared with male controls. Similarly, expression of *HULC* was higher in total GBS cases compared with controls and in male cases compared with male controls. Similar pattern of expression was detected between total cases and total controls. *HULC* has been shown to regulate immune responses through miR-128-3p/RAC1 axis^26^. In line with our observations, miR-128-3p has been shown to be down-regulated in cerebrospinal fluid of animal models of GBS^27^. RAC1 regulates a number of inflammatory pathways such as STAT3 and NF-κB^28^. NF-κB pathway has a documented effect in the pathogenesis of immune-related neuropathies^29^. Therefore, HULC/miR-128-3p/RAC1 axis might also been involved in the pathogenesis of CIDP and GBS.

*PVT1* was up-regulated in CIDP cases compared with controls and in both male and female CIDP cases compared with sex-matched controls. Similarly, *PVT1* was up-regulated in GBS cases compared with controls and in total patients compared with total controls. Contrary to this finding, we have previously reported down-regulation of *PVT1* in the peripheral blood of patients with multiple sclerosis^30^. Therefore, this lncRNA might have distinctive effects in these two inflammatory conditions.

Expression levels of *DSCAM-AS1* and *SPRY4-IT1* were higher in CIDP and GBS cases compared with controls and in both sexes compared with sex-matched controls. Therefore, these lncRNAs have a consistent pattern of expression among CIDP and GBs patients potentiating them as biomarkers for these conditions.

Although *LINC-ROR* was up-regulated in total CIDP and total GBS cases compared with controls, in sex-based comparisons, it was only up-regulated in male CIDP cases compared with male controls indicating the possible interactions between this lncRNA and sex-related parameters, since there was no gender-based difference in phenotype of the patients in terms of severity of illness.

Finally, expression of *MEG3* was up-regulated in all subgroups of patients versus controls except for male GBS controls. Expression of *MEG3* has been shown to be elevated in CD4 + T cells of patients with immune thrombocytopenic purpura. Expression of this lncRNA has been reduced in CD4 + T cells cultured with dexamethasone^12^. Functionally, *MEG3* inhibits Foxp3 expression and increases RORγt expression, thus inducing imbalance between regulatory T cells and Th17 cells^12^. The imbalance between these subsets of T cells might participate in the pathogenesis of GBS or CIDP as previous studies have shown the therapeutic effects of regulatory T cells in animal models of GBS^31^.

The correlations between expression levels of mentioned lncRNAs were not meaningfully different between patients and controls based on the measured correlation coefficients. *SPRY4-IT* and *DSCAM-AS1* could differentiate CIDP cases from controls with appropriate diagnostic power values. Similarly, these lncRNAs had high power in differentiation between GBS cases and controls. Since expression levels of lncRNAs were almost similar between CIDP cases and GBS cases, none of lncRNAs could appropriately differentiate between CIDP and GBS cases. Combination of all lncRNAs could not significantly enhance the diagnostic power. Taken together, these lncRNAs might be involved in the development of CIDP or GBS. These transcripts might be regarded as marker for these immune-related conditions as well. Future studies should appraise expression of these transcripts in other immune-related conditions to evaluate their suitability as diagnostic markers for GBS/CIDP.

## Data Availability

The datasets used and/or analyzed during the current study are available from the corresponding author on reasonable request.
